# Establishment of Animal Model of Dual Liver Transplantation in Rat

**DOI:** 10.1371/journal.pone.0040818

**Published:** 2012-07-19

**Authors:** Ying Zhang, Yong He, Raaj Kumar Praseedom, Shusen Zheng, Jiahong Dong, Hao Chen

**Affiliations:** 1 Department of Microbiology, Chinese PLA General Hospital, Beijing, China; 2 Department of Molecular Genetics and Microbiology, College of Medicine, University of Florida, Gainesville, Florida, United States of America; 3 Key Laboratory of Combined Multiorgan Transplantation, First Affiliated Hospital, College of Medicine, Zhejiang University, Hangzhou, Zhejiang, China; 4 Department of General Surgery, NIHR Comprehensive Biomedical Campus, Addenbrookes Hospital, Cambridge, United Kingdom; 5 Hospital & Institute of Hepatobiliary Surgery, Chinese PLA General Hospital, Beijing, China; 6 Department of General Surgery, Zhejiang Province People’s Hospital, Zhejiang, China; 7 Department of Cardiology, College of Medicine, University of Florida, Gainesville, Florida, United States of America; University of Colorado School of Medicine, United States of America

## Abstract

The animal model of the whole-size and reduced-size liver transplantation in both rat and mouse has been successfully established. Because of the difficulties and complexities in microsurgical technology, the animal model of dual liver transplantation was still not established for twelve years since the first human dual liver transplantation has been made a success. There is an essential need to establish this animal model to lay a basic foundation for clinical practice. To study the physiological and histopathological changes of dual liver transplantation, “Y” type vein from the cross part between vena cava and two iliac of donor and “Y’ type prosthesis were employed to recanalize portal vein and the bile duct between dual liver grafts and recipient. The dual right upper lobes about 45–50% of the recipient liver volume were taken as donor, one was orthotopically implanted at its original position, the other was rotated 180° sagitally and heterotopically positioned in the left upper quadrant. Microcirculation parameters, liver function, immunohistochemistry and survival were analyzed to evaluate the function of dual liver grafts. No significant difference in the hepatic microcirculatory flow was found between two grafts in the first 90 minutes after reperfusion. Light and electronic microscope showed the liver architecture was maintained without obvious features of cellular destruction and the continuity of the endothelium was preserved. Only 3 heterotopically positioned graft appeared patchy desquamation of endothelial cell, mitochondrial swelling and hepatocytes cytoplasmic vacuolization. Immunohistochemistry revealed there is no difference in hepatocyte activity and the ability of endothelia to contract and relax after reperfusion between dual grafts. Dual grafts made a rapid amelioration of liver function after reperfusion. 7 rats survived more than 7 days with survival rate of 58.3.%. Using “Y” type vein and bile duct prosthesis, we successfully established a novel rat model of dual right upper liver lobe transplantation.

## Introduction

Liver transplantation is an effective treatment for end-stage liver disease, but a huge gap remains between the number of people that need a liver transplant and the number of organs available. In order to maximize donor organs, novel surgical techniques such as split liver and living donor transplantation have evolved [1]. However, small for size syndrome is a major issue with single lobe liver grafts both in split and live donor liver transplantation. Animal models of whole orthotropic rat liver transplantation [2] and small-for-size liver transplantation [3] have been established to study this syndrome. Dual liver transplantation in clinical practice happens sporadically and there exists a significant gap in our knowledge regarding the physiological changes in these patients. In spite of the development of dual liver transplantation in Korea, China, and Japan (Table 1) [4]–[8], animal models to evaluate dual liver transplantation are still not to be established. Here we report the first rat model of dual liver transplantation.

**Table 1:** Dual liver transplantation history.

Country	Number	Donor graft	Graft ratio	Time	Author	University
China	2	1 Dual LL 1 RL+LL	1.5% GRWR 1.2% GRWR	2006	Wang[^4^] wentao	West ChinaHospital, Sichuan University
Germany	2	1 LLS+LL1 LLS+RL	46% to 54%. GV/SLV	2006	Dieter[^5^]	University Hospital of Hamburg-Eppendorf
Korea	17	12 dualLL4 LLS+LL1 RL+LL	46.6% to 78.9%. GV/SLV	2000.3–2001.7	S G lee[^6^]	Asan Medical Center, College of Medicine, Ulsan University
Korea	2	1 DualLLS1 LL+LLS	1.4 GR/WR 0.69 GR/WR	2006	DeokBog Moon[^7^]	University of Ulsan College of Medicine
Japan	1	RL+LL	65.4 GV/SLV	2008	Y Soejimaa[^8^]	Sapporo Medical School Hospital University of Tokushima School of Medicine

LL left lobe, RL right lobe, LLS left lateral segment, GRWR grafts to recipients’ weight ratio. GV/SLV graft volume/standard liver volume.

## Results

### Microcirculation

**Figure 1:**
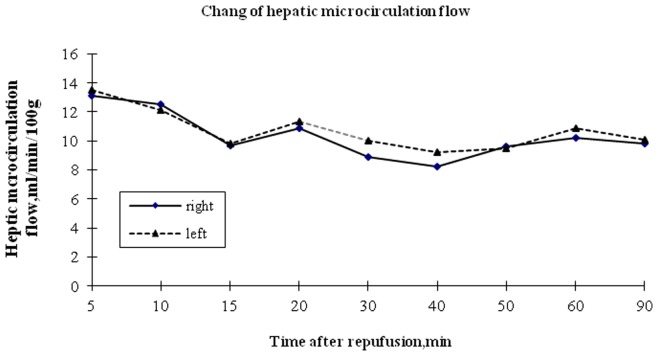
Change of hepatic microcirculation flow. Laser Doppler probe showed that no difference in microcirculation flow between orthotopically implanted graft and heterotopically positioned graft (p>0.05).

The hepatic microcirculatory flow in orthotopically implanted graft and heterotopically positioned graft were 10.7±1.01 ml/min/100 g and 11.2±1.21 ml/min/100 g respectively. No significant difference was found between two grafts (p>0.05) (Figure 1).

### Light Microscope

**Figure 2:**
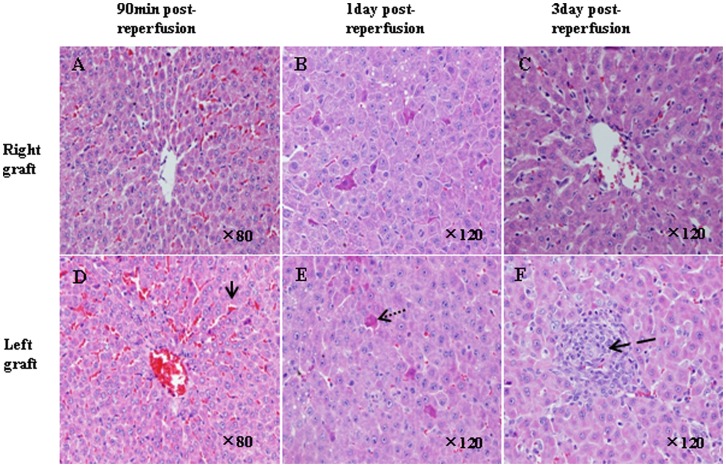
Histology results by HE staining. A and D, Ninety minutes after reperfusion: right graft showed moderate sinusoidal congestion morphological changes(A), left graft shows moderafte-severe sinusoidal congestion and vacuolar changes in the cytoplasm of hepatocytes (D). B and E, 1 day after reperfusion: apoptosis of 3–4 hepatocyte in the right and left graft was seen. C and F, 3 days after reperfusion: normal liver architecture was found in right graft (C), and scatter necrosis can be seen in left graft(F), H&E staining magnification×80 for A and D, ×120 for B,C,E and F. The normal arrow pointed to the sinusoidal congestion, the dot arrow indicated the hepatocyte apoptosis(eosino-dying), the broken arrow displayed the necrosis.

In orthotopically implanted graft, the hepatocytes and portal tracts showed moderate sinusoidal congestion morphological changes. In 3/12 heterotopically positioned graft, obvious sinusoidal congestion and vacuolar change in the cytoplasm of hepatocytes 90 minute after reperfusion was found, scatter necrosis was found 5 days after transplantation in 2/12 heterotopically positioned graft (Figure 2).

### Electron Microscopy

**Figure 3:**
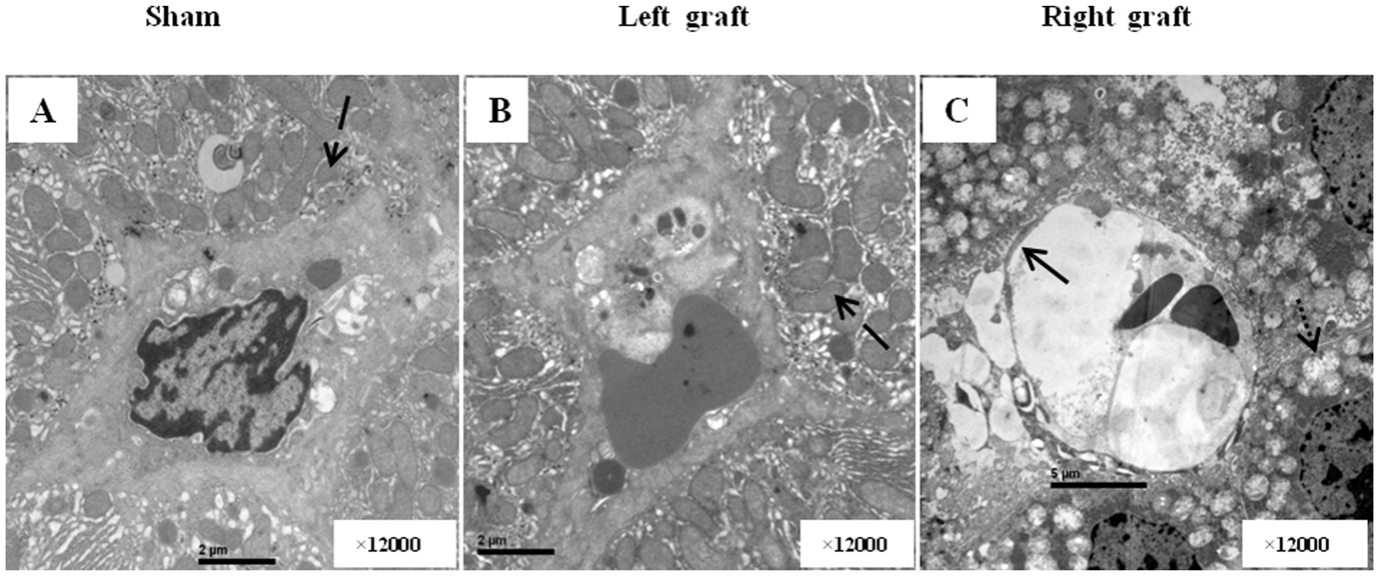
Electron microscopy results 1 day after reperfusion. A, In sham group, hepatocyte and sinusoidal cell had normal appearance. B, In right graft, The continuity of the endothelium was preserved except for minimal edema and defenestration. the liver architecture was maintained without obvious features of cellular destruction. C In left graft, integrity of endothelial cells was disrupted. Sinusoidal congestion and irregular large gap in the sinusoid lining cells were found. Mitochondrial swelling and vacuolar changes in cytoplasm of hepatocytes were obvious. The normal arrow indicated the endothelium, the dot arrow indicated mitochondrial vascuolar changes.

In orthotopically implanted graft, the liver architecture was maintained without obvious features of cellular destruction. The continuity of the endothelium was preserved except for minimal edema and defenestration. There was no evidence of progressive cell damage. Only 3 heterotopically positioned graft showed the patchy desquamation of endothelial cell 90 mins after reperfusion, mitochondrial swelling and hepatocytes cytoplasmic vacuolization were at 1 day after reperfusion (Figure 3).

### Immunohistochemistry

**Figure 4:**
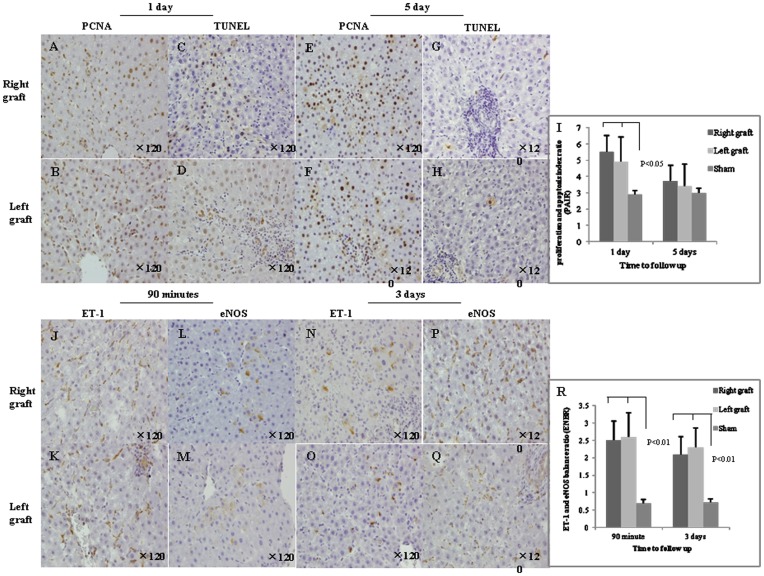
Proliferation and apoptosis of Hepatocyte and contraction and relaxation of endothelium by immunostaining. A and B, PCNA staining in right and left grafts 1 day after reperfusion. C and D, TUNEL staining in right and left grafts 1 day after reperfusion. E and F, PCNA staining in right and left grafts 5 day after reperfusion. G and H, TUNEL staining in right and left grafts 1 day after reperfusion. I, Proliferation and apoptosis index ratio was significantly increased in right and left graft 1 day after reperfusion (p<0.05), compared to the sham group. No difference was found between right and left grafts. J and K, ET-1 staining in right and left grafts 90 minutes after reperfusion. L and M, eNOS staining in right and left grafts 90 minutes after reperfusion. N and O, ET-1 staining in right and left grafts 3 days after reperfusion. P and Q, eNOS staining in right and left grafts 3 days after reperfusion. R, ET-1 and eNOS balance ratio in right and left grafts were dramatically increased 90 minutes and 3 days after reperfusion compared to the sham group (p<0.01). No difference was found between right and left grafts. Brown stained cell is positive cells. ET-1, endothelin-1; eNOS, endothelial NO-synthase; ENBR, ET-1 and eNOS balance ratio; PAIR, proliferation and apoptosis index ratio; PCNA, proliferation cell nuclei antigen; TUNEL, terminal deoxynucleotidyl transferase-mediated nick-end labeling.

In order to evaluate the hepatocyte activity to undergo proliferation and apoptosis after reperfusion, proliferation and apoptosis index ratio (PAIR, ratio of proportion of PCNA and TUNEL decorated hepatocellular nuclei) was used as the weighing parameter which can reflect priority inclination of cell to proliferation and (or) apoptosis. At 1 and 5 day after reperfusion, PAIR in the orthotopically implanted graft (5.5±1.01, 3.7±0.98) was higher than that in the heterotopically positioned graft (4.9±1.54, 3.9±1.35) without significant difference. The PAIR in dual grafts is higher than that in sham group 1 day after reperfusion (p<0.05) (Figure 4).

The ability of endothelia to contract and relax after reperfusion was judged through the ET-1 (Endothelin-1) and eNOS (Endothelial Nitric Oxide Synthase) balance ratio (ENBR) which is proportion of immunostaining intensity score timed positive cell number score of ET-1 to that of eNOS. 90 mins and 3 days after reperfusion, grafts displayed an increased immunoreactivity of ET-1, whereas the eNOS protein expression was reduced with an ENBR 2.5±0.56, 2.1±0.51 in the orthotopically implanted graft respectively, 2.6±0.69, 2.3±0.56 in the heterotopically positioned graft respectively. The significant difference in ENBR was not found between the orthotopically implanted graft and the heterotopically positioned graft. Compared to the sham group, the ENBR is higher than those in dual graft 90 minutes and 3 day after reperfusion (p<0.01) (Figure 4).

### Serum Parameters

Preoperatively, all serum parameters were within the normal range (Table 2). Dual graft led to a maximum of ALT release of 577.4±101.6 U/l on the third day and then significantly reduced to 310.2±57.2 U/l on the 5th day after reperfusion. Albumin levels dropped on the first day to 2,108.1±108.4 mg/dl recover to 2,369.7±226.5 U/l on the 5th day after reperfusion. Bilirubin levels after reperfusion were gradually increased, it was 52.4±19.9 U/l on the 5th day.

**Table 2:** Serum parameters.

Parameter	Group	Preoperatively	90 m after surgery	1^st^ postoperatively	3^nd^ postoperatively	5^th^ postoperatively
ALT (<40 U/I)	DRU	26.5±4.1	392.1±146.8^a^	534.4±185.1^a^	577.4±101.6 a	310.2±57.2^a^
	SHAM	22.2±1.8	28.3±4.3	41.9±9.6	34.6±4.9	28.9±4.1
Albumin (2700–3000 mg/dl)	DRU	2,780±89.6	2,516.7±98.4	2,108.1±108.4 a	2,288.8±180.1 a	2,369.7±246.5
	SHAM	2,714.3±90	2,628.6±95.1	2,771.4±95.0	2,718±141.1	2,785.7±110.9
Blirubin (<1.0 um/L)	DRU	7.6±1.7	17.9±9.8^b^	32.0±13.8^b^	37.8±16.5^b^	52.4±19.9^a^
	SHAM	7.4±1.6	7.5±1.7	7.7±1.8	7.6±1.6	7.4±1.4

a *P*<0.01 DRU versus SHAM,

b *P*<0.05 DRU versus SHAM,

(DRU: dual right upper lobe, ALT, alanine aminotransferase).

### Survival Rate

**Figure 5:**
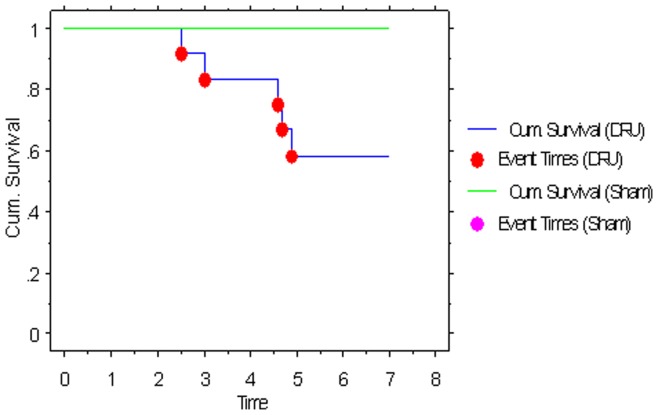
Survival analysis in animal model of dual liver transplantation . In sham group, all mice (n = 12 ) survived more than 7 days. On 7 mice in dual right upper lobe transplantation survived more than 7 days with survival rate of 58.3%. DRU indicated the dual right upper lobes of liver.(long rank test could not get the p values because the in sham group contain no uncensored observation).

12 rats were used to do survival analysis. 7 rats survived more than 7 days with survival rate of 58.3%. Abdominal bleeding, bile leakage, liver abscess in graft was found in 5 dead rats (Figure 5).

## Discussion

The shortage of liver donors has put the patients at risk of dying on the waiting list for liver transplantation. Although new surgical techniques such as split liver transplantation and living donor liver transplantation play an important role in extending the donor pool, it can not be routinely carried out, especially when graft weight and (or) volume can not meet the basic needs of recipient and when graft quality is marginal which can lead to small-for-size syndrome or primary non-function [9]. Dual liver transplantation has been effective in dealing with potential small for size syndromes. Several countries have put this technique into practice and obtained satisfactory results. However, the small number of cases and the short follow-up resulted in a paucity of information regarding the physiological processed in dual liver transplantation. An animal model of dual liver transplantation is therefore urgently required to provide a strong foundation for scientifically based clinical practice.

The optimal graft volume to provide adequate functional hepatocytes is still a controversial issue. At present, there are two standards worldwide: one is ratio of grafts to recipients’ weight (GRWR) and the other is ratio of grafts volume to recipients’ standard liver volume (GV/SLV). It is generally thought that the former should be more than 0.8% [10], and the latter should be more than 40% [11]. In the present animal study, the weight of dual right upper lobe was about 45–50% of recipient’ liver.

Presently and clinically, dual left lobe or one left and one lateral segment graft or one left and one right lobe graft or two left side (lateral segment or left lobe) grafts was employed in dual liver transplantation. A left lobe from a relatively small volunteer donor will not meet the metabolic demands of a larger recipient. The possible solutions to this problem are to increase the extent of resection in the donor by harvesting the right lobe of the liver, which accounts for 60% to 70% of the total liver mass, to apply auxiliary partial orthotopic liver transplantation, or to implant dual grafts into one recipient. The right lobe harvest in the donor is not always safe, depending mainly on the volume of the remaining left lobe. Even though the donor has sufficiently large right lobe that is adequate as a graft for an adult recipient, the remaining left lobe may be too small to bring a safety to donor in many occasions. In this instance, the donor cannot be allowed to donate either side of the liver lobe for a large-size adult recipient. As an alternative, dual left lobe or left lateral segment from two living donors can solve the problem of graft-size insufficiency and guarantee donor safety. Furthermore, if the large-size recipient requires a bigger graft liver volume than the volume of the sum from two potential living donors’ left lobes, and if the right lobe harvested from one of two potential donors seems to be safe, one right lobe and one left lobe from two donors can be transplanted into a single recipient to avoid a small-for-size graft problem. In the present animal study, two right upper lobes were used as dual liver grafts. There were two reasons for using two right upper lobes as grafts. Firstly, if the left or middle lobe was used as a graft, in order to preserve the vena cave a large part of the residual right lobe would also need to be taken due to the vena cava going through whole right lobe. Secondly, the total volume of two right upper lobes gives 45–50% of total recipient liver weight which is a more appropriate size for the recipient.

Difficulties in dual liver transplantation lie in the anastomosis of suprahepatic inferior vena cava, portal vein and bile duct between the graft and the recipient. Although two-cuff technique has been used in animal models of single liver transplantation, it can not be put into effect in dual liver transplantation due to the double suprahepatic inferior vena cava, double portal vein and double bile duct anastomosis of the grafts to a single surahepatic inferior vena cava, portal vein and bile duct in the recipient. To overcome these problems we use the “Y” type vein from donor and Self-developed “Y” type bile prosthesis. “Y” type vein is the cross part where vena cava is divided into left and right iliac vein. This arrangement that two cuff of left and right iliac were inserted into portal vein of dual graft and the cuff of recipient’ portal vein were inserted into the vena cava of “Y” type vein made the blood flow direction as the original “Y” type vein did without vortex. (Figure 2). The recanalization of the bile duct was carried out with an interposion of the “Y” type prosthesis between the donor and the recipient grafts. Previously we tried to insert catheter attached bile duct of orthotopically implanted graft into the bile duct of recipient, and do the anastomosis of heterotopically positioned graft’ bile duct and duodenum which resulted in the higher incidence of liver abscess and bile leakage because the bacteria reflex without the protection of sphincter of Oddi. The usage of “Y” type prosthesis solves this problem.

It was postulated that excessive portal blood flow relative to small-for-size graft and insufficient arterial perfusion would induce portal hypertension and arterial hypotension, which in turn lead to injury of the graft. Since the maximum additional hepatic blood flow volume that the liver can tolerate is 20% [1], Portal hypertension and excessive portal blood flow can result in sinusoidal injury from sinusoidal congestion and disruption of sinusoidal lining cells. Hepatic sinusoidal cells play a critical role in the maintenance of hepatocyte function, under both physiological and pathological conditions. The homeostasis of hepatic microcirculatory environment is crucial for the early recovery of graft function after reperfusion [12]. The dual graft developed mitochondrial swelling, sinusoidal congestion and large gaps in sinusoidal lining cells at 90 minute after reperfusion, but the liver architecture recovered without causing any conspicuous endothelial damage except for minimal cellular edema and defenestration.

It is interesting to speculate the reasons which might protect the liver architecture from progressive damage. The balance of ET-1/eNOS plays an important role. Under physiological conditions, the sinusoidal diameters are regulated by a balance between ET-1 and NO produced from L-arginine, e.g. by the constitutive eNOS [13], [14]. The balance is changed with the alteration in graft size. ET-1 and eNOS balance ratio (ENBR) was used to evaluate the imbalance between contract and relax of endothelia in our study. At 90 minutes after reperfusion there was a significant increase of ET-1 and decrease of eNOS as the ENBR in dual grafts was significantly higher than that in shamed liver. It might be postulated that rapid onset of ET-1 as well as eNOS reduced activity is a compensative reaction to over-perfusion. Compensation in hemodynamics can potentially lead to better functional recovery of hepatocytes and higher survival rate. At 1 days after reperfusion, PAIR, reflecting the ability of cell to proliferation and apoptosis, in dual grafts is significantly higher than that in shamedliver. Similarly, the levels of ALT, Albumin and Bilirubin recovery quickly.

Because one of the dual graft is heterotopically positioned and rotated 180° sagitally, theoretically the change of spacial position potentially lead to the difference of hepatocyte shear force. Although morphological test display that 3 heterotopically positioned graft showed obvious sinusoidal congestion, vascular change in the cytoplasm of hepatocytes, scatter necrosis and patchy desquamation of endothelial cell, no significant differences in microcirculation, cell activity and the ability of endothelia to contract and relax were observed between orthotopically implanted graft and heterotopically positioned graft which means that right upper lobe can be rotated 180° sagitally as a heterotopically positioned donor.

In the present study, although using ‘Y’ type vein and ‘Y’ type bile prosthesis we successfully established a rat model of dual liver transplantation, there is still some limitation:1) the dual donor are both right upper lobe which is different form the combination in clinical practice; 2) the operation time, cool ischemia time and anhepatic phase is longer; 3) the requirement for microsurgical skill is higher because of complicated anastomosis of vena cava, portal vein and bile duct, Usually at least two surgeon need to complete this operation; 4) the 7 day accumulative survival is only 58.3%, so still need to try hard to make a improvement.

In the future, we will use this animal model to explore mechanism underlying the cooperative regeneration in dual graft, competitive regeneration resulting in one graft atrophy, the immune microenviroment change between dual graft and between recipient and dual donor and study on implication of marginal donor as dual grafts.

## Materials and Methods

### Animal and Study Design

Male Sprague-Dawley rats were used as donors and recipients. Rats were housed in a standard animal laboratory with free activity and access to water and chow. They were kept under constant environmental conditions with a 12-hour light-dark cycle. The rats were fasted 12 hours before operation. All the operations were performed under clean conditions.

**Figure 6:**
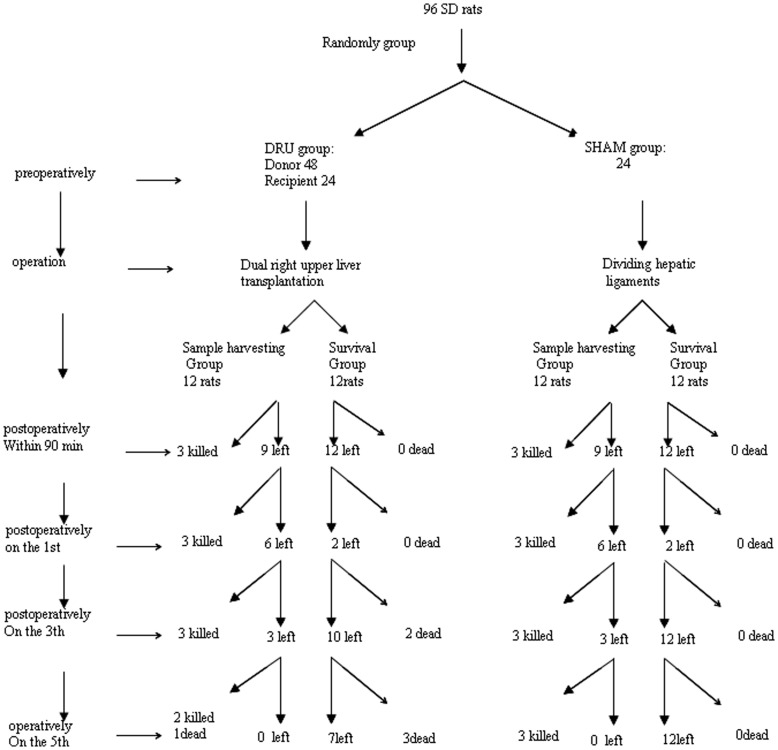
Experiment group and animal number.

The protocol of animal experiments was approved by the Institutional Ethical Committee of Animal Experimentation of Zhejiang University in china, and the experiments were performed strictly according to governmental and international guidelines on animal experimentation. According to requirements for Biosafety and Animal Ethics ‘Units and individuals who are conducting the production and use of experimental animal production, should treat animals humanely and protect animal welfare, should not tease and abuse animals. The use of experimental animals should be in accordance to the scientific, rational and humane requirements. It is advised and encouraged to minimize the use of laboratory animals to reduce suffering of animals to be disposed of, and to explore of alternative methods in replacing animal testing and use’, Every effort was made to minimize any suffering of the animals used in this study. The isogeneic male SD (male, 8–10 weeks, 231.95±20.9 g, Shanghai Animal Center, Chinese Academy of Science, Shanghai) we used in our study among which 48 rats were taken as donors, 24 as recipients, dual right upper lobes (dual upper portion of right lateral lobe, 45–50% of the recipient liver weight ) were planted in the one recipient. 12 recipients were assigned to for specimens harvest and undergo hemodynamic, morphological and serum monitoring, another 12 recipients were kept under observation up to the 7th postoperative day for survival analysis. In sham group (n = 24), the liver was mobilized by dividing the hepatic ligaments. 12 rats in sham group were assigned for specimen harvest and the remaining 12 rats for survival analysis (Figure 6).

The graft weight relative to the liver weight of recipient was determined after the recipient liver was harvested. Whole operation time, cold ischemic time, warm ischemic time and anhepatic phase were calculated (table 3).

**Table 3:** Graft, donor and recipient data.

Parameter	dual liver graft
Graft hepatic lobe	Dual right upper lobe
Graft weight(gm)	4.71±0.68
Recipient liver weight(gm)	9.5±0.98
Donor weight(gm)	232.6±21.1
Recipient weight(gm)	231.3±19.8
GRLWR	45–50%
Operation time(minute)	75
CIT(minute)	32
WIT(minute)	13
Anhepatic phase(minute)	23

GRLWR grafts to recipients liver weight ratio,

CIT cold ischemic time, WIT warm ischemic time.

### “Y” Type Vein and Bile Duct Prosthesis

**Figure 7:**
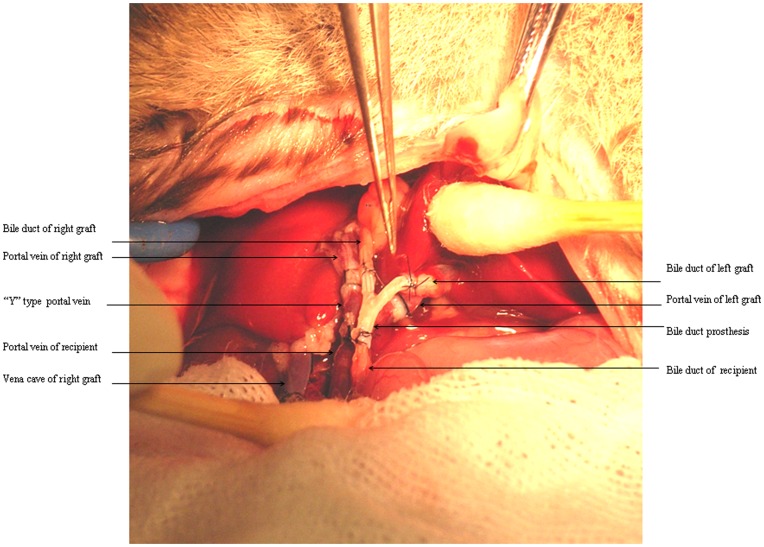
Y type vein and bile duct prosthesis in vivo.

In order to recanalize portal vein and the bile duct between dual liver grafts and recipient, “Y” type vein and bile duct from donor were used and designed in this study. The branch of vena cava and iliac vein were ligated and then “Y” type part where vena cava is divided into left and right iliac vein was harvest. Two cuffs were made in left and right iliac vein in vitro. The vena cava of “Y” type vein was left to be inserted into by the cuff of recipient vena cava. The recanalization of the bile duct was carried out with an interposion of the “Y” type prosthesis between the donor and the recipient grafts. Two catheters inserted into bile duct of the dual grafts were inserted into the two upper leg of “Y’ type prosthesis respectively in vitro, the catheter inserted into the lower leg of the “Y” type was inserted into the recipient’ bile duct in vivo (Figure 7).

### Donor Operation

All procedures were carried out under nitrous oxide/isoflurane anaesthesia (N_2_O/O_2_ = 2∶1+1.5% isoflurane). Liver transplantation was performed on the basis of two cuffs technique described by Kamada N et al [15], [16]. All donor hepatolobectomies were carried out in vivo, taking care to protect the blood supply and avoiding damage to the inferior vena cava and portal vein. The common bile duct was inserted a polyethylene catheter. Donor livers were then harvested with a rapid perfusion of 8 ml of Ringer’s balanced solution through a catheter placed in abdominal aorta. The left lobe, the median lobe, two caudate lobes and right dower lobe were separately removed by ligation with #5-0 silk sutures to achieve right upper lobe which was selected for dual liver transplantation. Dual right upper lobes of the liver add up to the 45–50% weight of recipient liver. The isolated graft was put in a container filled with ice-cold saline. For dual liver donors, the first graft is designed to orthotopically implanted at the original position and the second graft is heterotopically positioned in the left upper quadrant rotated 180° sagitally. The orifices of the 2 supra hepatic cava were reconstructed in order to obtain a single site for anastomosis in the recipient. The infrahepatic vena cava of the first graft were prepared and inserted into polyethylene cuffs for implantation. The infrahepatic vena cava of the second graft was ligated. The second donor was selected to ligate the branch of vena cava and iliac vein and harvest the “Y” type part where vena cava is divided into left and right iliac vein, and left and right iliac vein were inserted into polyethylene cuffs and then the two cuffs were inserted into the portal vein of dual graft separately in vitro. After the dissociation, the bile duct was incised one the anterior wall, and inserted into a small drainage catheter with an internal diameter of 0.6 mm, secured with a circumferential 5-0 silk ligature.

### Recipient Operation

The recipient and two donor operations are started simultaneously by three surgeons. The first liver graft is orthotopically implanted at its original position (right graft). Then the second liver graft is heterotopically positioned in the left upper quadrant and rotated 180° sagitally (left graft). The rotation of the heterotopic second liver graft through 180° sagitally brings the hilar structures into a reversed position. Thus the bile duct comes to lie behind the portal vein. The anastomosis of the suprahepatic vena cava was performed using continuous #8-0 polypropylene sutures between the recipient’s suprahepatic and the reconstructed suprahepatic vena cava of the dual liver donors. The portal anastomosis was performed using the ‘Y’ type vein, two cuffs of two iliac vein of “Y” type vein were already inserted into the recipient’s portal vein when repairing the graft in vitro. The portal vein of recipient was inserted into a cuff which was inserted into the vena cava of “Y” type vein in vivo. The recanalization of the bile duct was carried out using the ‘Y’ type prosthesis. Two catheters inserted into bile duct of the dual grafts were already inserted into the two upper leg of “Y’ type prosthesis respectively in vitro. The catheter attached the lower leg of the “Y” type was inserted into the lumen of recipient’ bile duct. Anastomosis of infrahepatic vena cava of the first donor was completed by the cuff method and the infrahepatic vena cava of the left graft was ligated (Figure 7).

After the completion of the surgical procedure, recipient animals were cared for under an intensive post operative recovery protocol.

### Microcirculation

Microcirculation of the liver graft was measured with laser Doppler (BPM^2^ Blood Perfusion Monitor, Laserflo BPM^2^; Vasamedics, St Paul.Min). A laser Doppler probe (model P-440 Soflex Implantable probe, Laserflo BPM^2^, Vasamdics) was placed on the surface of the right upper lobe of the graft. Satisfactory contact of the probe on graft surface was maintained during the blood flow detection. Hemodynamic data were analyzed using the PowerLab software system (PowerLab System, ADInstruments Pty Ltd).

### Hematoxylin and Eosin Stain

For histological evaluation, all liver specimens were fixed by immersion for at least 1 day in 10% buffered formaldehyde phosphate and were subsequently dehydrated and embedded in paraffin wax to cut sections at a thickness of 4 um. All tissue specimens were cut from both grafts. Staining was performed as routine procedure. Sections were evaluated for proliferation and apoptosis of the hepatic parenchyma. Morphologic parameters were recorded by routine histology.

### Electron Microscopy

Ultrastructural parameters were recorded by scanning electron microscopy. Liver specimens were cut into 2×2×5 mm^3^ blocks, ethanol-dehydrated, freeze-fractured in liquid nitrogen, prepared critical point-dried, ion-sputtered, coated with platinum and viewed with a high-resolution scanning electron microscope (1530 VP, LEO, Germany). Photographs were taken at a magnification of 12,000.

### Immunohistology and TUNEL (Terminal Deoxynucleotidyl Transferase-mediated Nick-end Labeling)

For immunohistochemical staining a HRP/DAB detection IHC kit (ab80436 Abcam, Cambridge, MA, USA) was used per the manufacturer’s protocol. Deparaffinize and rehydrate formalin-fixed paraffin-embedded tissue section. Add Hydrogen Peroxide Block to cover the sections and incubate for 10 minutes. After antigen retrieval (antigen retrieval buffer, ab64236. Abcam, America) for 20 min in a domestic pressure cooker and after blocking non-specific binding sites with protein block, the sections were immunoreacted with primary antibodies (ET-1 1:50, rabbit polyclonal antibody sc-21625 Santa Cruz Biotechnology Inc, American; eNOS 1∶100 rabbit polyclonal antibody, ab66127 abcam, America; PCNA 1∶250 rabbit polyclonal antibody, ab18197, abcam, America) overnight at 4°C. Apply Rabbit specific HRP conjugate and incubate for 15 minutes at room temperature. DAB was applied to the tissue and sections counterstained with haematoxylin.

Sections were stained for apoptotic cells with a DeadEnd™ Colorimetric TUNEL System from Promega Corporation. Images shown are representative of at least three independent experiments which gave similar results. The proliferation and apoptosis index ratio (PAIR), i.e. the ratio of the proportion of TUNEL and PCNA decorated nuclei per 100 hepatocellular nuclei, was semi-quantitated by counting approximately 1,000 hepatocytes. The immunostaining intensity of eNOS and ET-1 was evaluated by a four-grade score: 0 = negative, 1 = partially (<25%) weak positive-stained cells, 2 = partially (<25%) moderate or diffuse (>75%) weak positive cells, 3 = diffuse (>75%) moderate or strong positive cells, 4 = diffuse (>75%) strong positive cells. The number of eNOS and ET-1 labeled cells was scored semi-quantitatively from 0 to 4 (0 = 0, 1 = 10–300, 2 = 310–600, 3 = 610–900, 4 = >900 stained cells in 10 observed areas per section). Calculate the ET-1 and eNOS balance ratio (ENBR), i.e. the ratio of staining intensity multiplied positive semi-quantitative number of ET-1 and eNOS.

### Serum Parameter

Preoperatively, 90 min after reperfusion and 1, 3 and 5 days after operation, alanine-aminotransferase (ALT), albumin and bilirubin were determined at 25°C by standard enzymatic techniques (micromethod, Ektachem-Kodak, Germany).

### Survival Analysis

12 recipients in each group were used for the survival study.

### Statistic Analysis

All data are presented as means±SD. Statistical analysis was performed by the t test and Kruskal–Wallis test using SPSS 13.0 software. Survival rates were assessed by the Kaplan–Meier method. The log-rank test was used to compare significance. A level of significance of *P*<0.05 was considered as sufficient in all experimental groups.
